# The Preparation of Hollow Mesoporous Bioglass Nanoparticles With Excellent Drug Delivery Capacity for Bone Tissue Regeneration

**DOI:** 10.3389/fchem.2019.00283

**Published:** 2019-04-26

**Authors:** Yudong Wang, Haobo Pan, Xiaofeng Chen

**Affiliations:** ^1^Research Center for Human Tissue and Organs Degeneration, Institute Biomedical and Biotechnology, Shenzhen Institutes of Advanced Technology, Chinese Academy of Sciences, Shenzhen, China; ^2^School of Materials Science and Engineering, South China University of Technology, Guangzhou, China; ^3^National Engineering Research Center for Tissue Restoration and Reconstruction, Guangzhou, China

**Keywords:** hollow structure, nanoparticles, bioglass, drug delivery, bone repair

## Abstract

In this work, hollow mesoporous bioglass (HMBG) nanoparticles were prepared in a hexadecyl trimethyl ammonium bromide (CTAB)-cyclonexane-ethanol-water (O/W) emulsion system. The HMBG nanoparticles possessed higher drug storage ability and stable drug release behavior which resulted from HMBG's unique mesoporous structure. The mesoporous structure could be modulated by adjusting the concentration of CTAB. The specific surface area and drug loading efficiency was as high as 749.619 m^2^g^−1^ and 55.1%. Besides, *in vivo* experiments demonstrated that the HMBG nanoparticles could promote the bone tissue regeneration and the drug-loading HMBG nanoparticles possessed better repair capability. The unique structure and properties might make the HMBG nanoparticles good candidates as drug carriers and repair materials for bone tissue regeneration.

## Introduction

In 1969, Larry L. Hench invented 45S5 Bioglass® (46.1 mol% SiO_2_, 26.9 mol% CaO, 24.4 mol% Na_2_O, and 2.5 mol% P_2_O_5_), which had been widely used in clinical bone repair (Hench, 1991; Jones, 2001). Bioglass possessed good osteoconductive, osteoproductive, and osteoinductive properties because of its chemical reactions occurring on the material surface (Hench and Polak, 2002; Jones, 2013). The released ions from bioglass contributed to a highly active apatite layer, up-regulation of osteogenic gene expression and stimulation of osteoblast proliferation and differentiation (Hench et al., 1998; Jones et al., 2001). Along with the development of sol-gel fabrication and the application of the organic template, BG particles of different shapes and sizes were prepared, which demonstrated better osteogenic property compared with the traditional melt-derived 45S5 bioglass (Hench and West, 1990; Lei et al., 2011;Hu et al., 2014; Wang et al., 2017).

However, the sole osteogenic capability of bioglass cannot satisfy all the clinical demand when treating some osteal symptoms (Fenton et al., 2018; Khan and Tanaka, 2018; Pugliese et al., 2018). For example, the partial chronic inflammation would retard the bone tissue regeneration in the later repair process (Vieira et al., 2017; Martin and Bettencourt, 2018; Pugliese et al., 2018). Besides, some cancer cells could not be completely eliminated in the tumor resection, which might lead to subsequent canceration, hence more treatments should be developed to maintain patients' health (Sercombe et al., 2015; Ohta et al., 2016; Lee et al., 2017; Li et al., 2017). As a result, more and more studies focused on the fabrication of drug delivery particles which possess peculiar mesoporous structure and good biocompatibility (Hao et al., 2015; Prokopowicz et al., 2016; Kang et al., 2018). Some researchers had successfully prepared bioglass particles with different mesoporous structures, while these particles were still faced with the problem of agglomeration and lower drug delivery capacity (Wang and Li, 2016; Chen et al., 2017; Tang et al., 2017). Bioglass particles with good osteogenic and drug delivery properties were needed to further regenerate the bone tissue.

The emulsion preparation has been applied in particle fabrication for many years while it was seldom utilized for bioglass fabrication. Emulsion was defined as an optically isotropic and thermodynamic stable system composed of two immiscible liquid phases (water and oil) and surfactants (Imhof et al., 1997; Dong et al., 2010; Li and Shi, 2014; Shen et al., 2014). A monolayer of surfactant molecules self-assembled at the interface between the droplets and the continuous phase; and the droplets could be tuned by the surfactant and phase composition (Klajn et al., 2007; Du and Qiao, 2015; Zhang et al., 2015; Xiong et al., 2016). In this work, we provided a facile synthesis of hollow mesoporous bioglass (HMBG) by combing the sol-gel method with the micro-emulsion technique. CTAB not only acted as the surfactant in the emulsion system but also as the template for the mesoporous structure. The HMBG nanoparticles were characterized and they demonstrated preferable ability of drug delivery and bone tissue regeneration.

## Materials and Methods

### Materials

Tetraethyl orthosilicate (TEOS), triethylphosphate (TEP), calcium nitrate tetrahydrate (CN), ethanol absolute (EtOH), and ammonia solution (25 wt % NH_3_ in water) were purchased from Guangzhou Chemical Reagent Factory. Hexadecyl trimethyl ammonium bromide (CTAB) and cyclohexane were supplied by Aladdin (Shanghai, P. R. China). All chemical reagents mentioned above were analytical grade. Deionized water was obtained from a water purification system (Milipore S.A.S, France).

### Preparation of HMBG Nanoparticles

The preparation of HMBG nanoparticles had been reported before (Wang and Chen, 2017). Briefly, 4.0 ml TEOS was dispersed in 75.0 ml cyclohexane by stirring to form an oil phase. The oil solution and CTAB were then added to a mixture of 190.0 ml deionized water and 110.0 ml ethanol. Thereafter, 2.0 ml ammonia solution was added into the solution to initiate the reaction at 40°C to promote TEOS's hydrolysis. The mixture was then stirred for 10 min, and 0.31 ml TEP and 1.70 g CN were sequentially added to the mixture every 30 min. After 3 h, the resulting products were collected by filtration, rinsed with ethanol and deionized water, and dried at room temperature for 24 h. Finally the HMBG nanoparticles were obtained after removing organics and nitrates by calcination at 650°C for 3 h. CTAB concentrations of 2, 4, and 6 mM were prepared for comparison and the corresponding HMBG nanoparticles were denoted HMBG-1, HMBG-2, and HMBG-3, respectively.

### Characterization of HMBG Nanoparticles

The samples' morphologies, microstructure, and particle size distribution were determined using the scanning electron microscope (SEM, MERLIN Compact, Carl Zeiss, Germany) and transmission electron microscopy (TEM, JEM-2100F, JEOL, Japan). Specific surface area was measured using the multipoint Brumauer–Emmett–Teller (BET) N_2_ absorption technique at 77.3 K. The pore width and distributions were calculated by the Density functional theory (DFT) based on the desorption isotherm.

### Studies of Drug Storage and Release

In this work, Ibuprofen (IBU) was chosen as the model drug to evaluate the drug storage and release ability of HMBG nanoparticles. 1.0 g HMBG was added into 50 ml IBU hexane solution (20 mg/ml) at room temperature. The vials were sealed to prevent the evaporation of hexane and the mixture was then stirred for 24 h. HMBG nanoparticles adsorbed with IBU were separated from the solution by centrifugation, washed with diluted HCl solution (pH 1.0) three times and dried at 60°C. Filtrates (1.0 ml) were extracted from the vials and diluted to 20 ml, and then analyzed by UV–vis spectroscopy (Tu-1901, Persee, Beijing, P. R. China) at a wavelength of 264 nm.

The calibration curve of ibuprofen was determined by taking absorbance vs. ibuprofen concentration between 0 and 200 mg ml^−1^ as parameters. For this interval, the calibration curve fits Lambert and Beer's law


(1) $$\begin{array}{r} A=0.034\times C+0.043 \end{array}$$

A was the absorbance and C was the concentration (mg ml−1). The samples storing IBU molecules were denoted as IBU-HMBG. Three parallel samples at one desired concentration of IBU hexane solution were examined.

IBU-HMBG samples were compacted into 0.1 g discs (diameter and thickness, respectively, 6^*^3mm) under a pressure of 4 MPa. A typical *in vitro* drug release experiment was performed as follows. The discs were immersed into the PBS solution at 37°C, under stirring at a rate of 100 r min^−1^. The release medium (2.0 ml) was removed for analysis at given time intervals using a syringe and replaced with the same volume of fresh preheated release medium. The 2.0 ml extracted medium was diluted to 20 ml with release medium and analyzed with UV–vis spectroscopy at a wavelength of 264 nm. Calculation of the corrected concentration of released ibuprofen is based on the following equation:


(2) $$\begin{array}{r} C_{tcorr}\;=\;C_{t}\;+\frac{v}{V}\overset{t-1}{\underset{0}{\sum }}C_{i} \end{array}$$

C_tcorr_ is the corrected concentration at time t, C_t_ is the apparent concentration at time t, ϑ is the volume of samples taken and V is the total volume of dissolution medium. Also, three parallel samples were tested *in vitro* drug release experiment.

### *In vitro* Bioactivity

The *in vitro* bioactivity of the obtained HMBG nanoparticles were tested by immersing in SBF (Na^+^ 142.0, K^+^ 5.0, Mg^2+^ 1.5, Ca^2+^ 2.5, Cl^−^ 147.8, ${\text{HCO}}_{3}^{-}$ 4.2, ${\text{HPO}}_{4}^{2-}$ 1.0 and ${\text{SO}}_{4}^{2-}$ 0.5mmol L^−1^) at a concentration of 1 mg/ml at 37°C to monitor the formation of hydroxyapatite (HA) on the surface of the samples. At particular points in time, the solids were filtrated, washed with deionized water, dried in air, and characterized using scanning electron microscopy (SEM, MERLIN Compact, Carl Zeiss, Germany), Fourier transform infrared spectroscopy (FT-IR, Nexus, Nicolet Co., USA) and powder X-ray diffraction (XRD, X'pert PRO, Panalytical, Netherlands) with Cu Ka (1.548 Å).

### Cell Seeding and Culture

Human osteoblast-like cells (MG-63) were purchased from the American Type Culture. MG-63 were cultured in high glucose Dulbecco's modified Eagle's medium (H-DMEM) with 10% (v/v) fetal bovine serum (FBS) and 1% (v/v) penicillin/streptomycin (P/S) in a humidified 5% CO_2_ atmosphere at 37°C. The culture medium was refreshed every 2–3 days. Cells were sub-cultured at 80% confluence and were used at passages 3–10.

Prior to cell seeding, the HMBG nanoparticles were high-temperature sterilized. Cells were seeded in a 24-well plate at a density of 3 × 104 cells per well. After 1 day of culturing, the sterilized HMBG particles were placed into wells at a concentration of 50, 100, and 500 μg/ml. The cellular behaviors of MG-63 were determined throughout the 14-day culture period.

### Cell Viability Evaluation

Cell viability was measured by quantitatively cell counting and qualitatively live-dead staining. Cell proliferation was determined using a Cell Counting Kit-8 (CCK-8, Dojindo Laboratories, Japan) following the protocol. Briefly, cells were harvested on days 1, 3, 5, and 7. After the removal of the media, the samples were incubated in H-DMEM medium containing 10% CCK-8 reagent at 37°C for 1 h. Cells cultured without HMBG nanoparticles were used as a control. The absorbance was measured ata wavelength of 450 nm using a micro-plate reader (Thermo 3001, USA). Six specimens for each cultured time point were tested and each test was performed in triplicate.

To further understand the adherence and growth behavior of MG-63, fluorescence microscopy (FM, 40FLAxioskop, Zeiss, Germany) was used after culturing for 1 and 3 days. For the FM assessment, MG-63 cells were stained using a live cell labeling kit (Cell Explorer, AAT Bioquest). The staining process was performed according to the kit instructions. In brief, after culturing for 1 and 3 days, the culture medium was replaced by the staining solution, incubated at 37°C for 1 h, followed by washing with PBS, and then observed by FM.

### ALP Activity Assay

The osteogenic differentiation of MG-63 cultured on the HMBG particles was assessed by measuring the ALP activity qualitatively and quantitatively using p-nitrophenyl phosphate substrate (pN) (ALP kit, Thermochem). At 7 and 14 days, the culture medium was removed, and the cell layers were then rinsed gently 3 times using PBS. In the qualitative assay, the cells were lysed, and then lysate was assayed with the hydrolysis of pN in the presence of ALP enzyme. This evaluation was performed according to the manufacturer's instructions (Sigma, USA), and the absorbance was measured at a wavelength of 405 nm using a micro-plate reader. Cells cultured without HMBG were used as a control to correct the absorbance values. In the quantitative assay, the lysates were transferred to a centrifuge tube and centrifuged for 10 min at 4°C (13500g). The supernatant was collected for further assay using an ALP assay Kit (Beyotime) following the manufacturer's instruction. After normalizing to the total protein content, the ALP activity was calculated from a standard curve. The total protein content was examined using a Pierce BCA protein assay kit (Thermo Scientific, USA) and the results were expressed as mmol of pN produced per milligram of protein. Three individual experiments were carried out and each sample was conducted in quadruplicate.

### *In Vivo* Evaluation and Histological Analysis

Ten-weeks old Wistar rats obtained from the Laboratory Animal Center, South China Medical College, were used in this study. The experimental protocols were approved by the Institutional Animal Care. Rat bone defect model was performed by anesthetizing Wistar rats with 10% chloral hydrate, and a bone defect of 3 mm diameter × 3 mm deep was created on the tibia using an electric trephine drill. A 40 mg amount of HMBG and IBU-HMBG particles mixed with phosphate buffer was used to fill the bone defects and the wounds were sutured. All rats were sacrificed after 4 weeks. The samples were harvested and fixed in 10% formaldehyde. The morphology of tibia was assessed using micro-computed tomography (micro-CT; Skyscan 1176, Kontich, Belgium). The micro-CT images were reconstructed using the Feldkamp convolution back-projection algorithm and segmented into binary images using adaptive local thresholding. The samples were decalcified by immersing samples into decalcifying fluid for 4 weeks. Then 5 μm thick sections were cut from the paraffin-embedded tissues to conduct the histological evaluation. Hematoxylin and eosin (H&E) staining and Masson's Trichrome staining were performed with sections from each sample.

### Statistical Analysis

All data were expressed as mean ± standard deviation (SD) and were analyzed using a Student's *t*-test. A *p* < 0.05 was considered statistically significant.

## Results and Discussion

### Properties of HMBG Nanoparticles

**Figure 1:**
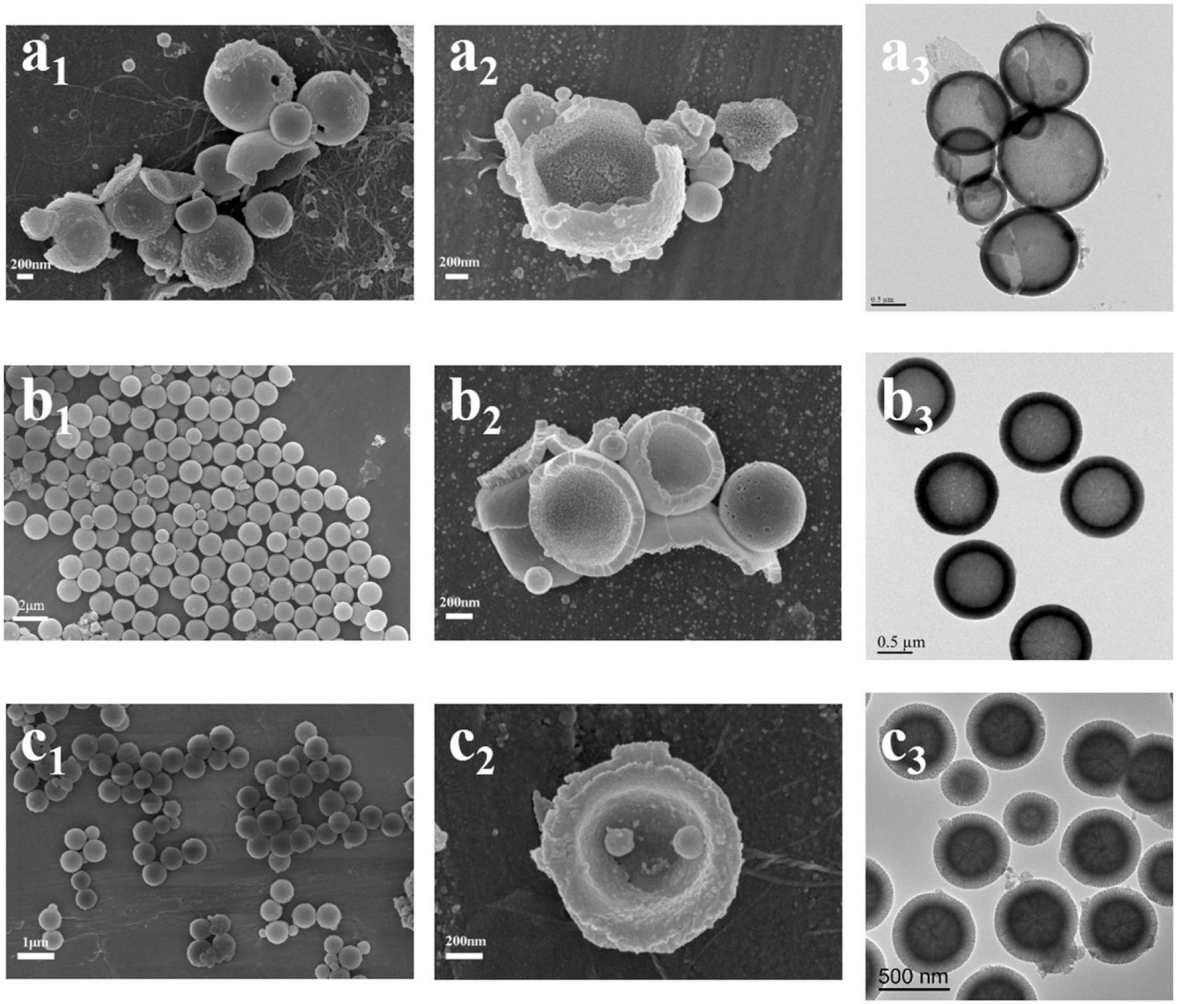
SEM and TEM images of HMBG-1 **(a)**, HMBG-2 **(b)**, and HMBG-3 **(c)**.

The morphologies and microstructure of HMBG nanoparticles under different CTAB concentration were characterized by SEM and TEM (Figure 1). HMBG nanoparticles under CTAB concentration of 2, 4, and 6 mM were denoted HMBG-1, HMBG-2, and HMBG-3, respectively. HMBG-1 possessed tanglesome morphology and severe agglomeration, as shown in Figures 1a1,a2, and part of the HMBG-1 nanoparticles were malformed. The TEM image demonstrated that HMBG-1 possessed hollow structure and the shell thickness was thin and dense compared with HMBG-2 and HMBG-3. The SEM images of HMBG-2 and HMBG-3 showed that both nanoparticles possessed good monodispersity and spherical morphology. The TEM images showed that HMBG-2 and HMBG-3 both possessed an obvious hollow structure and shell microstructure. Furthermore, HMBG-2 nanoparticles demonstrated much better monodispersity and size-unification.

**Figure 2:**
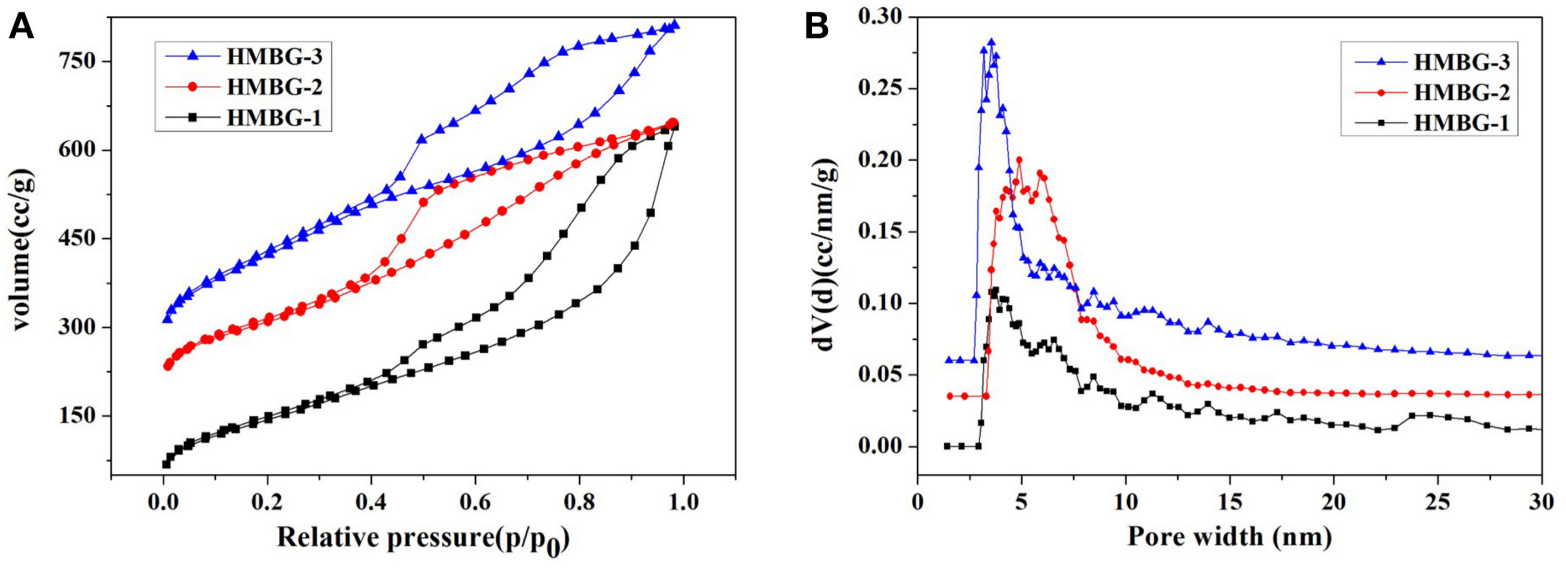
N_2_ absorption-desorption isotherm plots **(A)** and pore size distribution **(B)** of HMBG.

The specific surface area and pore structure of the HMBG samples were obtained by N^2^ absorption-desorption isotherm. As shown in Figure 2A, all samples exhibited type IV isotherm patterns with H3-type hysteresis loop associated with slot-shape mesopore, according to IUPAC classification (Coleman et al., 2000). The specific surface areas of HMBG-1, HMBG-2, and HMBG-3 were 540.400, 591.765, and 794.619m^2^g^−1^, respectively. As shown in Figure 2B, the pore structure of samples exhibited a narrow mesoporous size distribution. The average pore width of HMBG-1, HMBG-2 and HMBG-3 were 3.775, 4.887, and 3.537 nm, respectively. In the micelle system, micelles of different structure could be formed by adjusting the concentration of the template molecule (Hu et al., 2015; Luo et al., 2016). In this research, the pore structure was directly related to the micelle templates, which were formed by the accumulation of CTAB molecules. When the CTAB concentration was relatively low, more CTAB molecules contributed to the better stability and microstructure. However, the CTAB molecules would gather around the template micelle when increasing to a certain concentration, which had a bad influence on the formation of HMBG's mesopore structure and decreased the average pore width of HMBG.

### Drug Storage and Release Behavior

As mentioned above, the HMBG nanoparticles possessed a special hollow mesoporous structure and a large specific surface area, which made HMBG nanoparticles promising candidates for drug delivery and further application in bone tissue regeneration. The drug loading ability was calculated based on the UV–vis spectroscopy at the wavelength of 264 nm, shown in Table 1. The specific surface area and average pore width were also listed in Table 1, in order to make comparisons among HMBG-1, HMBG-2, and HMBG-3.

**Table 1:** The microstructure and drug loading ability of HMBG nanoparticles.

**Samples**	**Specific surface area (m^2^g^−1^)**	**Average pore width (nm)**	**Drug loading amount (mg)**	**Drug loading efficiency (%)**
HMBG-1	540.400	3.775	294.6	29.5
HMBG-2	591.765	4.887	489.6	49.0
HMBG-3	794.619	3.537	550.4	55.0

The table revealed that HMBG-3's specific surface area was much higher than HMBG-1 and HMBG-2's, which might have contributed to its higher drug loading efficiency. It was worth noting that HMBG-2 possessed favorable drug loading ability, as high as 49.0%, considering its lower specific surface area compared with HMBG-3.

**Figure 3:**
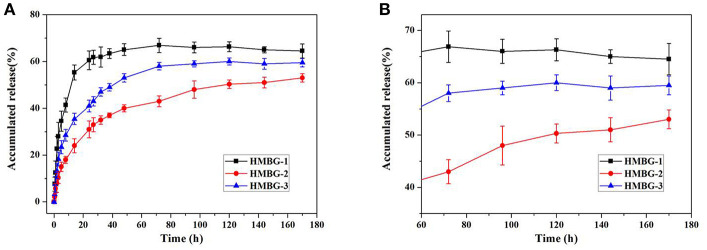
The drug release behavior of IBU-HMBG nanoparticles **(A)** and the magnifying image between 60h and 180h **(B)**.

Figure 3 showed the IBU release behavior of the drug loading HMBG nanoparticles in PBS solution. Three parallel *in vitro* drug release experiments were tested, and all data were expressed as means ± standard deviation (SD) for *n* = 3. In the earlier stage, some IBU molecules with slight adhesion were easily released from the surface of the IBU-HMBG nanoparticles, which demonstrated the burst release within 12 h. The release behavior of IBU-HMBG nanoparticles became slow and steady after 12 h interaction with PBS solution. After 60 h, IBU-HMBG-2 kept on releasing IBU molecules while the release amounts of IBU-HMBG-1 and IBU-HMBG-3 maintained at a certain degree. It was clear that IBU-HMBG-2 possessed an everlasting and steady drug releasing behavior compared with the other samples, which might result from its peculiar mesoporous structure.

**Figure 4:**
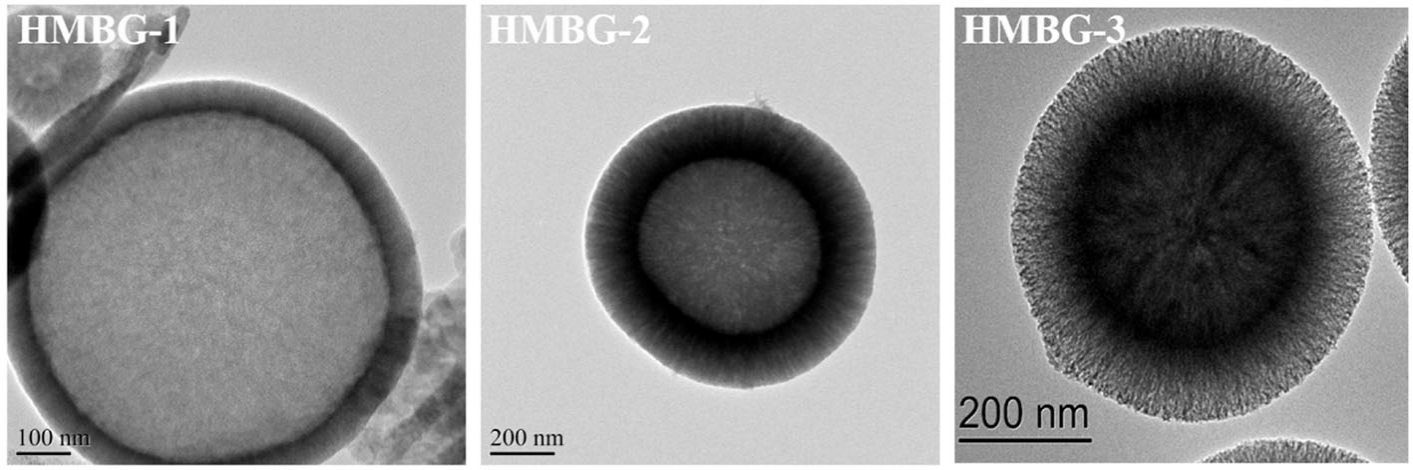
The TEM images of HMBG nanoparticles.

Figure 4 shows the TEM images of HMBG nanoparticles in large magnification; it was demonstrataed that all of the HMBG samples possessed an obvious hollow structure, while different samples have different shell property, which could have a great impact on their drug release behaviors. The shell of HMBG-1 was compact and the drug molecule could only be adhered to the shell surface. The HMBG-2 possessed a peculiar microstructure and the shell was composed of many orderly penetrative tunnels, which might transfer the drug molecules into the interior hollow part and ensure IBU-HMBG-2's sustained drug release behavior. The shell of HMBG-3 was composed of many mesoporous tunnels, which were similar to HMBG-2. Compared with HMBG-2, the mesoporous structure of HMBG-3 was tanglesome and the average pore width was much smaller than HMBG-2 (Table 1), which prevented the drug molecules from penetrating into the interior hollow part of HMBG-3 nanoparticles.

Combination of the above analysis, HMBG-2 was selected as the dominating material for the subsequent study.

### *In vitro* Apatite-Forming Ability of HMBG Nanoparticles

**Figure 5:**
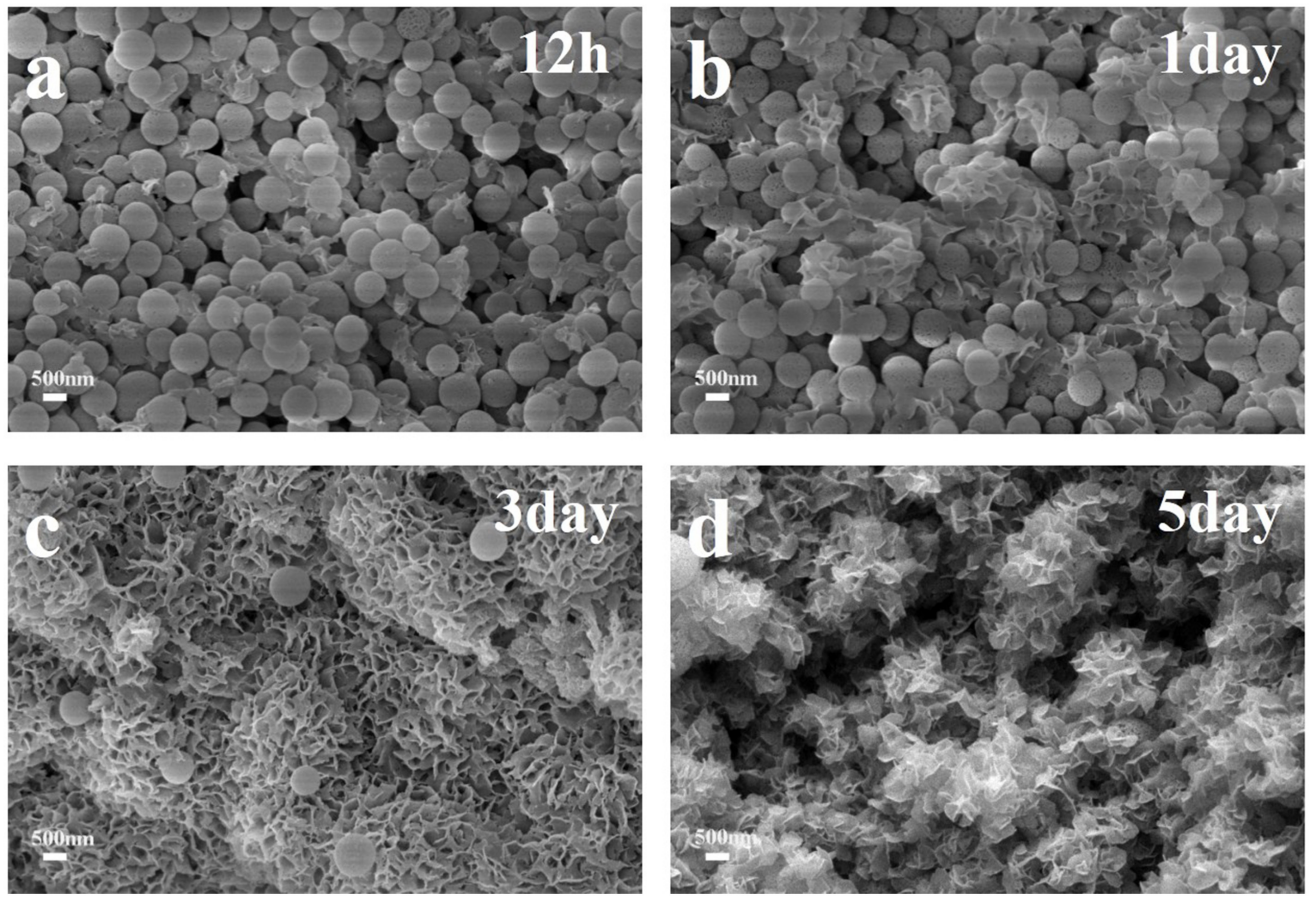
SEM micrographs of HMBG after soaking in SBF for 12h **(a)**, 1d **(b)**, 3d **(c)** and 5d **(d)**.

The apatite-forming ability has always been a measurement standard and an important property of bioactive glasses, which played a significant role in the bone tissue regeneration. In our experiments, HMBG nanoparticles were immersed in SBF for several time periods to evaluate the particles' apatite-forming ability. The SEM images of HMBG immersed in SBF for 12 h, 1, 3, and 5 days were shown in Figure 5 to analyze the morphology of the formed apatite. After soaking in SBF for 12 h, the surface of HMBG nanoparticles became coarser, covered by flaky apatite precipitates. With the increased soaking time, more flaky precipitates were formed in day-1. The flaky precipitate continued to grow, and the samples' surface grew the flower-like precipitates layer in day-3. After 5 days, the flower-like precipitates layer almost covered all the HMBG nanoparticles' surface.

**Figure 6:**
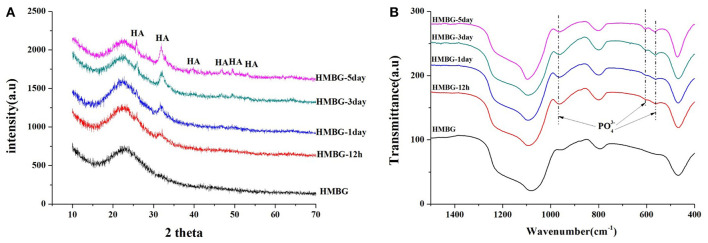
XRD patterns **(A)** and FT-IR spectra **(B)** of HMBG after soaking in SBF for different times.

The precipitates were confirmed to be the hydroxyapatite (HA) crystal according to the XRD and FT-IR results (Figure 6). The crystalline structure of the precipitation on the surface of the HMBG nanoparticles was demonstrated by XRD analysis (Figure 6A). Before soaking, only one small wide peak at around 24° was detected, indicating the amorphous nature of HMBG. After soaking, new peaks were observed at 2θ = 26°(002), 32°(211), 39°(310), 46°(222), 49°(213), and 53°(004), which corresponded to the crystallinity of HA (JCPDS 09-0432) (Ostomel et al., 2006). The FT-IR spectra of the samples, before and after soaking in SBF, were summarized in Figure 6B. Before soaking, the spectrum of HMBG showed characteristic absorption bands corresponding to Si-O-Si bonding at 1060 cm^−1^ (stretch vibration), 798 cm^−1^ (bending vibration), and 480 cm^−1^ (bending vibration). After soaking in SBF, a double band at 562 cm^−1^ and 603 cm^−1^, corresponding to the P-O bending vibrations of the phosphate group in a crystalline environment could be observed. The presence of absorption bands of the phosphate group at 968, 603, and 562 cm^−1^ demonstrated the deposition of HA on the surface of HMBG nanoparticles (Notingher et al., 2003). It was evident that the HMBG nanoparticles possessed good apatite-forming ability based on the analysis of SEM, XRD, and FT-IR.

### Cell Biocompatibility and Differentiation

**Figure 7:**
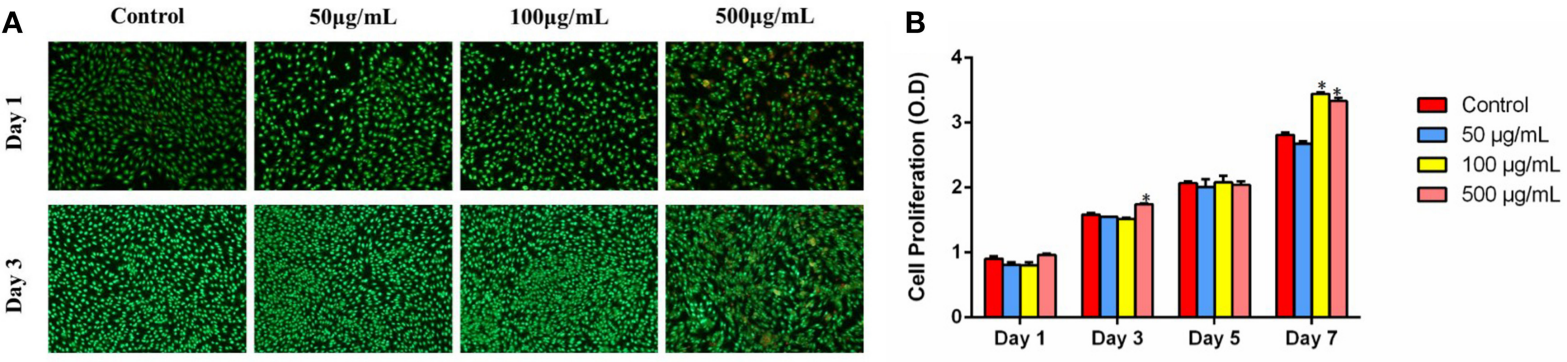
**(A)** FM images of MG-63 cells co-culturing with HMBG nanoparticles for 1 and 3 day. **(B)** Cell proliferation co-culturing with HMBGs particles at different culture times. *Statistically significant, *p* < 0.05 vs. control.

As could be seen from the FM images in Figure 7A, it was evident that MG-63 cells could spread well and exhibit good viability in all groups after being cultured for 1 day. With the increase in culturing time, the quantities of the live cells of all samples obviously increased, and few dead cells were detected in 500 μg/ml group.

Cell proliferation co-culturing with HMBG nanoparticles was evaluated using the CCK-8 test during 7 culturing days. As shown in Figure 7B, it was obvious that cells presented stable proliferation ability in all groups as the culture time increased. Cells in 500 μg/ml group showed slightly higher proliferation ability than 50, 100 μg/ml and the control group at the culture time of 3 days. Up to 7 days, MG-63 cells displayed significantly higher proliferation ability on 100 and 500 μg/ml groups, compared to the control. The results suggested that HMBG nanoparticles possessed good biocompatibility and could promote MG-63 cell proliferation greatly.

**Figure 8:**
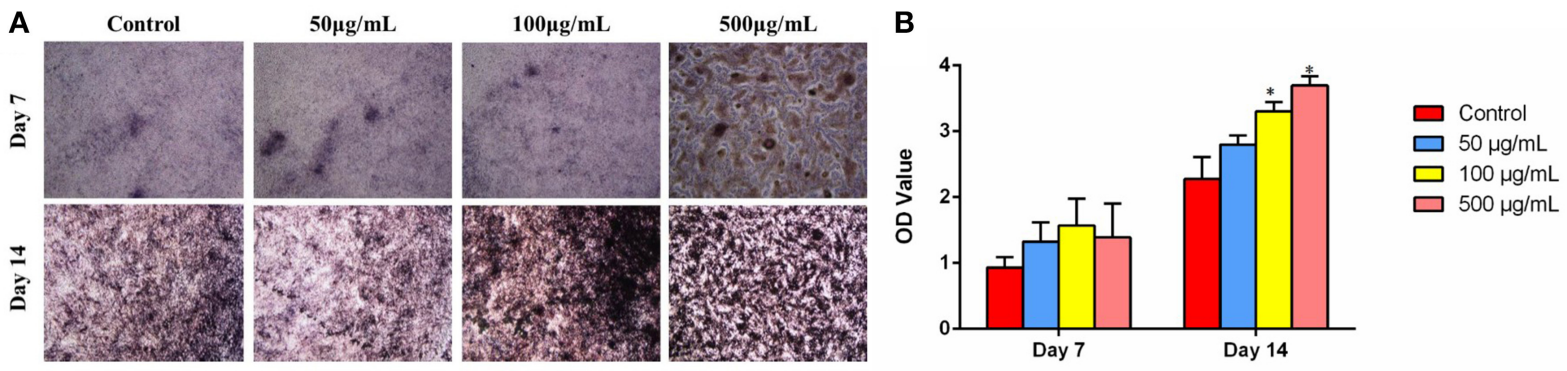
**(A)** ALP staining and **(B)** ALP activity expression of MG-63 cells after co-culturing with HMBG nanoparticles for 7 and 14 days. *Statistically significant, *p* < 0.05 vs. control.

Cell differentiation is one of the key processes for bone regeneration. It is known that ALP activity is an obvious osteogenic mark indicating cell differentiation ability. To investigate the effects of HMBG concentration on osteogenic differentiation of MG-63 cells, the ALP staining was performed on the 7 and 14th day after culturing with HMBG particles of different concentrations. As shown in Figure 8A, ALP staining results indicated that 50 and 100 μg/ml groups had higher osteogenic differentiation ability compared with the control group at the very initial stage (7 days), and then further promoted the differentiation process when the culture time prolonged to 14 days. In the 500 μg/ml group, the high concentration of HMBG had caused cell death partially after 7 days while HMBG nanoparticles could still promote the osteogenic differentiation after 14 days. Similar trends could be detected in the quantitative assessment of osteogenic differentiation as shown in Figure 8B. HMBG nanoparticles of the 100 and 500 μg/ml groups demonstrated significantly higher differentiation ability compared with the control group after 14 days. Based on the data above, we found that HMBG nanoparticles could induce the osteogenic differentiation of MG-63 cells prominently.

### *In vivo* Osteogenesis Ability of HMBG/IBU-HMBG Nanoparticles

**Figure 9:**
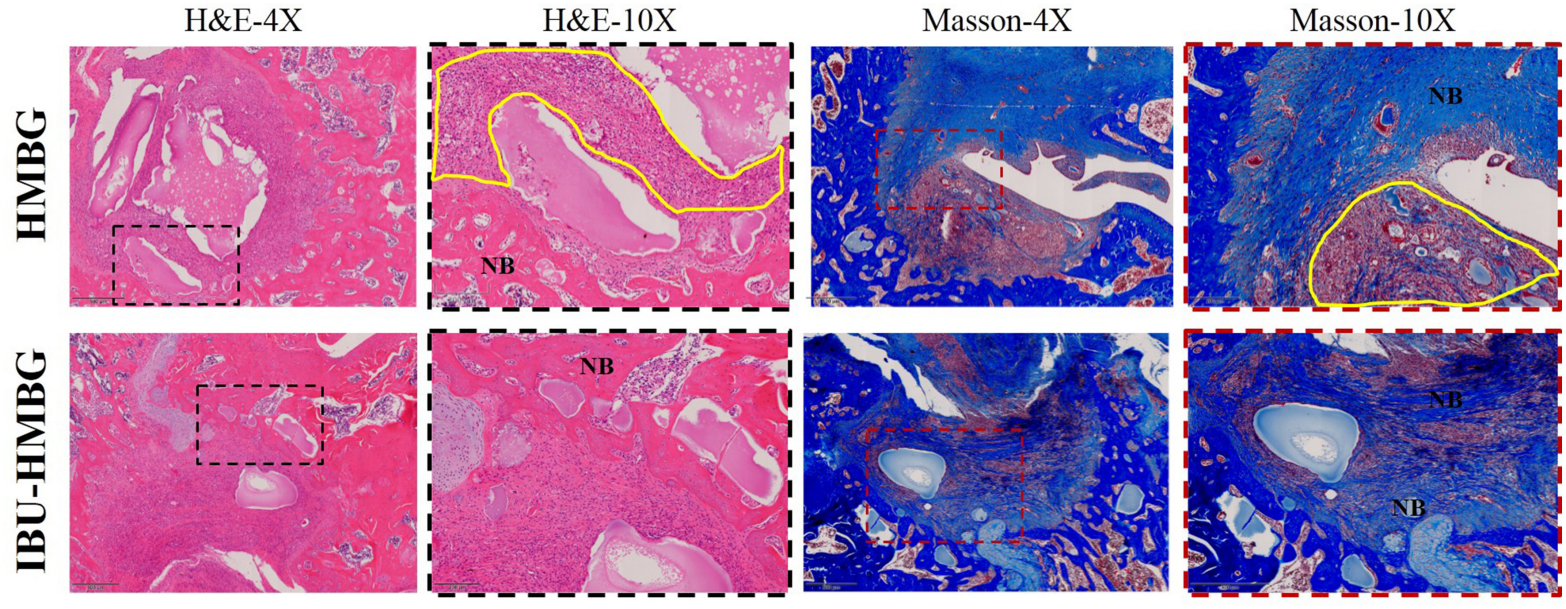
HandE and Masson's trichrome staining of HMBG and IBU-HMBG samples after 4 weeks. NB, new bone tissue; yellow circle, inflammation tissue.

**Figure 10:**
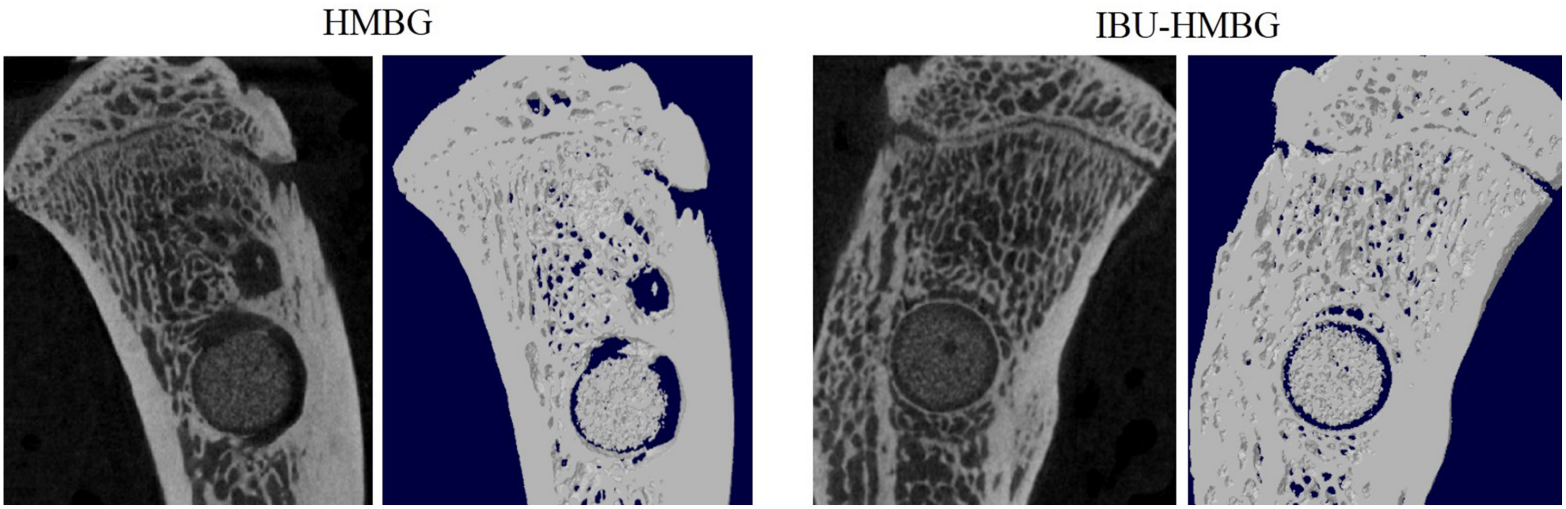
Micro-CT evaluation of bone regeneration in the rat tibia defects with implantation of HMBG and IBU-HMBG samples after 4 weeks.

HMBG and IBU-HMBG samples were implanted into the bone defects to evaluate the osteogenesis ability by the method of H&E and Masson's trichrome staining. As illustrated in Figure 9, new bone (NB) tissue was formed in both the HMBG group and IBU-HMBG group after 4 weeks' implantation. By comparison, H&E staining revealed that more new bone tissue was formed in the IBU-HMBG nanoparticles group and more inflammatory cells were found in the HMBG group, which proved the anti-inflammation of IBU-HMBG samples. It was also demonstrated by the Masson's trichrome staining that IBU-HMBG could promote osteogenesis and greatly relieve inflammation. Figure 10 showed the three-dimensional reconstructed images and sagittal section images of rat tibia with implantation of HMBG and IBU-HMBG composite for 4 weeks. Both groups demonstrated favorable bone remodeling ability and more new bone was found in the IBU-HMBG group.

Combining the data above, HMBG nanoparticles could promote osteogenesis while the drug-loading sample IBU-HMBG nanoparticles could better facilitate bone regeneration and alleviate local inflammation at the same time.

## Conclusions

Bioglass nanoparticles with hollow mesporous structure were prepared by combining the micro-emulsion fabrication and sol-gel technology. CTAB played an important role in modulating the interior mesoporous structure, morphology, and dispersity of HMBG nanoparticles. HMBG nanoparticles possessed higher drug loading and sustained drug release ability because of their special hollow structure and penetrative mesopores on the shell. HMBG nanoparticles loaded with ibuprofen demonstrated better bone tissue regenerative ability *in vivo* and the novel structure and property of HMBG nanoparticles might turn them into good candidates as drug carriers for bone tissue regeneration.

## Ethics Statement

Our study involved *in vivo* evaluation and histological analysis of biomedical material. Ten weeks old Wistar rats obtained from Laboratory Animal Center, South China Medical College, were used in this study. The experimental protocols were approved by the Institutional Animal Care of South China Medical College.

## Author Contributions

YW designed all the experiments, performed the preparation of HMBG nanoparticles and wrote the manuscript. HP provided the guidance on the biological characterization and XC gave the instruction during the material preparation process.

### Conflict of Interest Statement

The authors declare that the research was conducted in the absence of any commercial or financial relationships that could be construed as a potential conflict of interest.
