# Corrigendum: The Preparation of Hollow Mesoporous Bioglass Nanoparticles With Excellent Drug Delivery Capacity for Bone Tissue Regeneration

**DOI:** 10.3389/fchem.2022.726845

**Published:** 2022-03-09

**Authors:** Yudong Wang, Haobo Pan, Xiaofeng Chen

**Affiliations:** ^1^ Research Center for Human Tissue and Organs Degeneration, Institute Biomedical and Biotechnology, Shenzhen Institutes of Advanced Technology, Chinese Academy of Sciences, Shenzhen, China; ^2^ School of Materials Science and Engineering, South China University of Technology, Guangzhou, China; ^3^ National Engineering Research Center for Tissue Restoration and Reconstruction, Guangzhou, China

**Keywords:** hollow structure, nanoparticles, bioglass, drug delivery, bone repair

In the original article, there was a mistake in Figure 1 as published. The panel b1 was reproduced from a previous article without the appropriate attribution. The corrected Figure 1 appears below.

**FIGURE 1:**
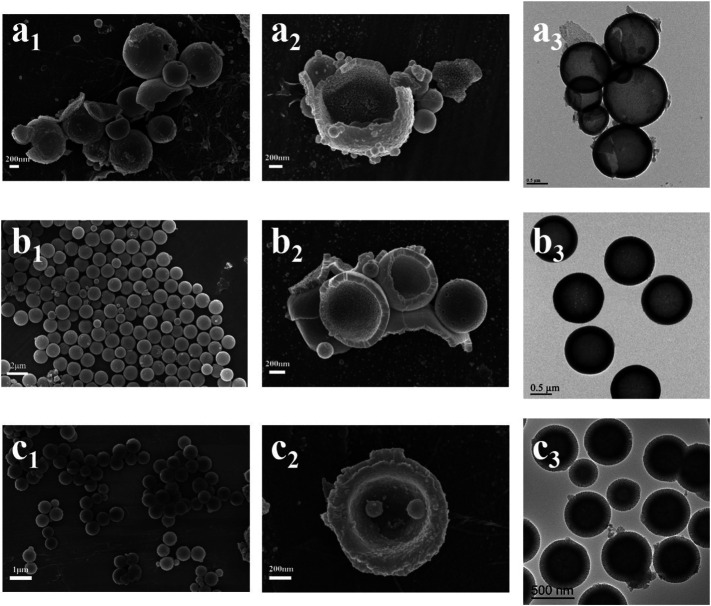
SEM and TEM images of HMBG-1 **(A)**, HMBG-2 **(B)**, and HMBG-3 **(C)**.

In the original article, there was a mistake in Figure 4 as published*.* Two panels in this figure (HMBG-1 and HMBG-2) were reproduced from a previous article without the appropriate attribution. The corrected Figure 4 appears below.

**FIGURE 4:**
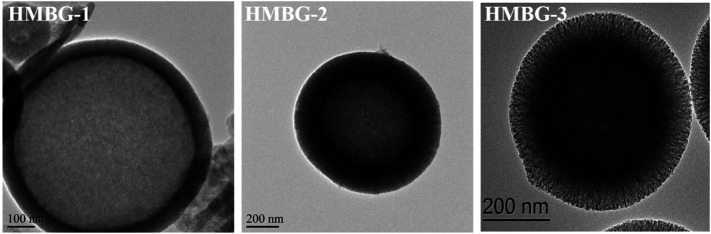
The TEM images of HMBG nanoparticles.

The authors apologize for this error and state that this does not change the scientific conclusions of the article in any way. The original article has been updated.

